# Supplementary material for: Sororin actively maintains sister chromatid cohesion
Source: EMBO J. 2016 Feb 22;35(6):635–53. doi: 10.15252/embj.201592532 (PMC4801952; doi:10.15252/embj.201592532)
Supplement: Supplementary file 1 — Appendix [file EMBJ-35-635-s001.pdf]

Sororin actively maintains sister chromatid cohesion

# - Appendix-

Table of contents:

Appendix Figures S1-S5………………………………………………….. Pages 2-6

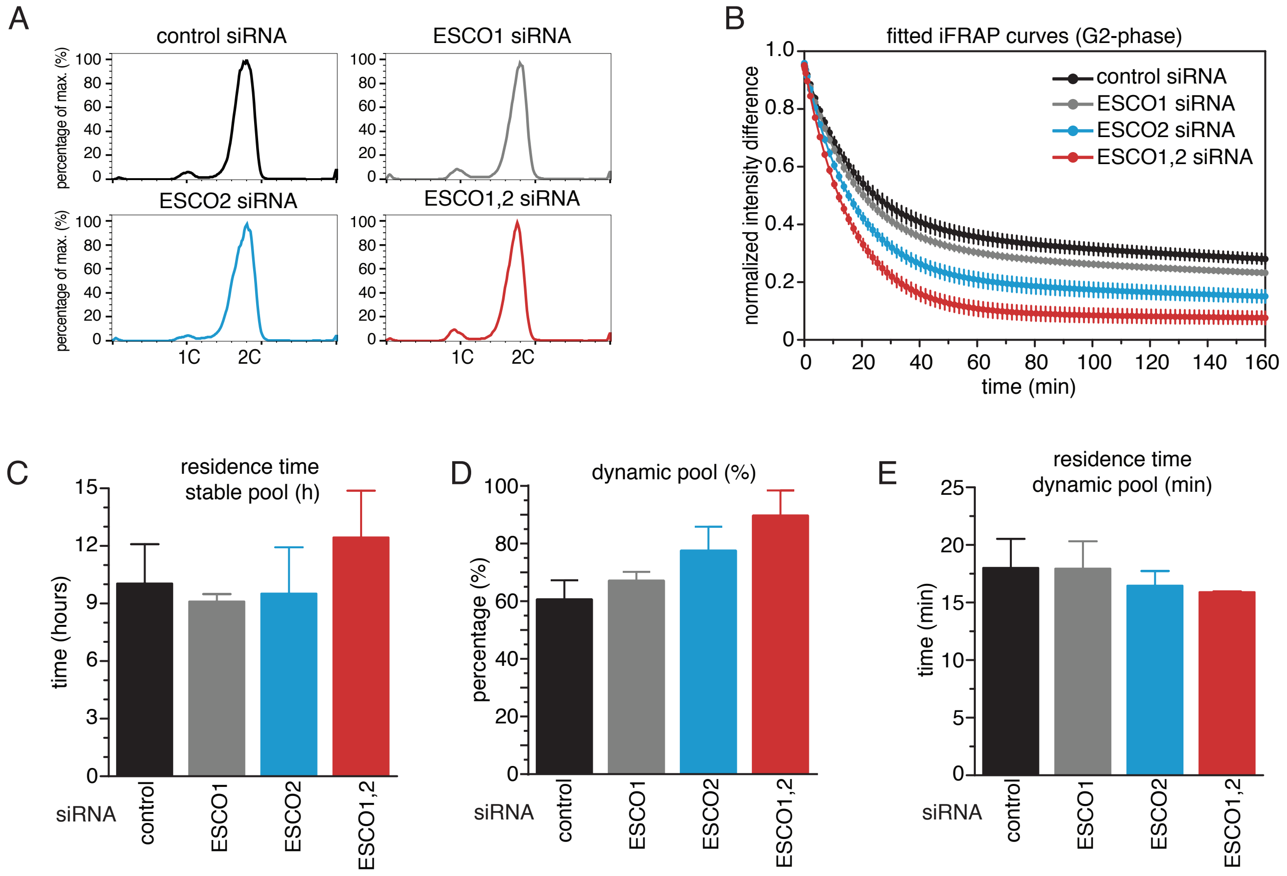

Appendix Figure S1. ESCO1 and ESCO2 are required for stable association of cohesin with chromatin (related to Fig 1)

A FACS profile of propidium iodide stained cells in Fig 1. Cells epxressing SMC3-LAP cells were synchronized in G2-phase and transfected with siRNA as indicated.

B Graph depicting iFRAP curves after fitting data in Fig 1C. Error bars denote s.e.m., n=10 cells per condition.

C Quantification of chromatin residence time of SMC3-LAP in the stable chromatin binding mode. Error bars denote s.e.m, n=10 cells per condition.

D Quantification of the relative abundance of SMC3-LAP in the dynamic chromatin binding mode. Error bars denote s.e.m, n=10 cells per condition.

E Quantification of chromatin residence time of SMC3-LAP in the dynamic chromatin binding mode. Error bars denote s.e.m, n=10 cells per condition.

Ladurner, Kreidl et al. Figure S2

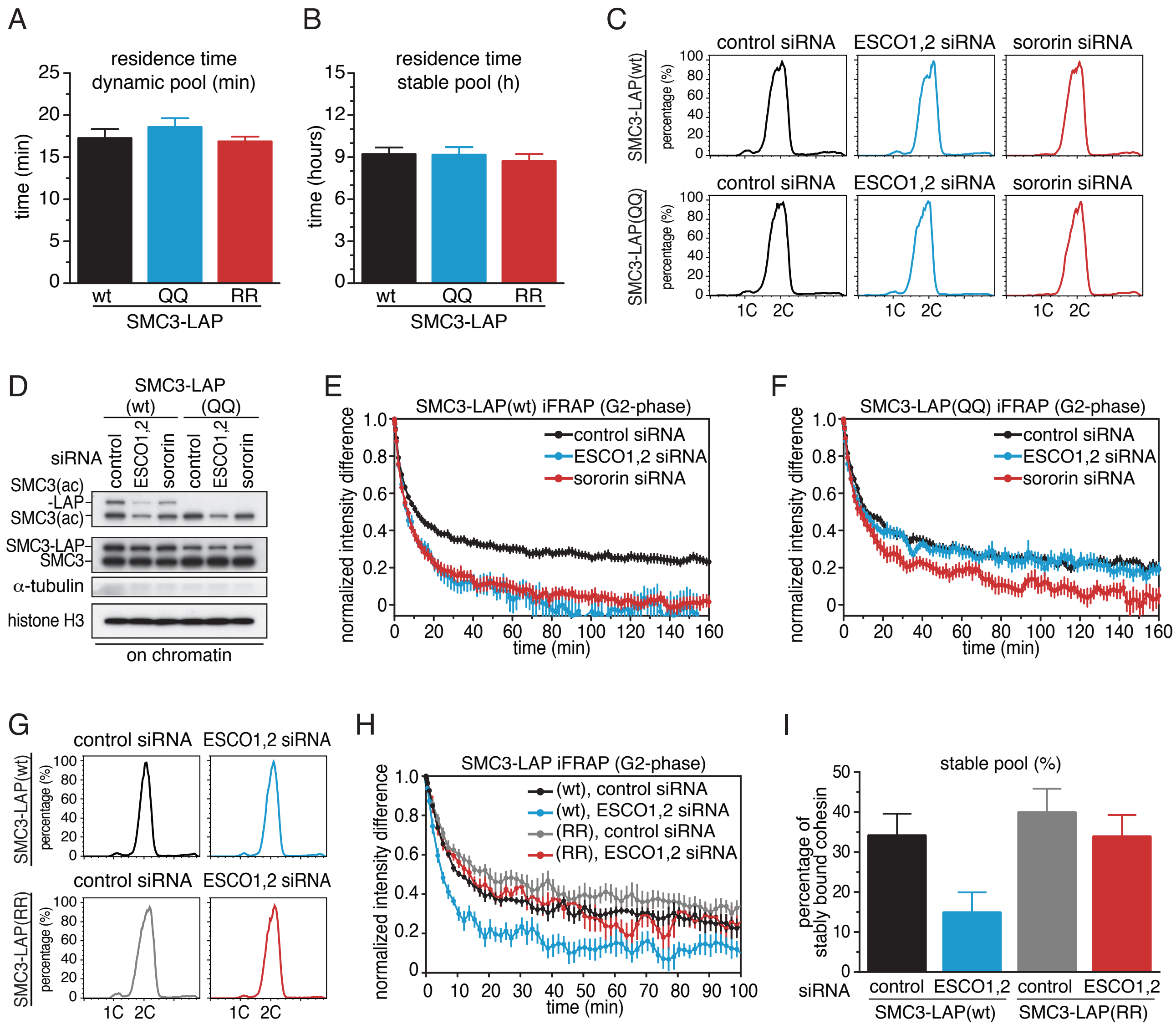

Appendix Figure S2. SMC3 acetylation site mutants generate stable cohesin-chromatin interactions without ESCO1 and ESCO2 (related to Fig 2)

A Quantification of chromatin residence time of G2-phase SMC3-LAP alleles in the dynamic chromatin binding mode (Fig 2C). Error bars denote s.e.m, n>26 cells per condition.

B Quantification of chromatin residence time of G2-phase SMC3-LAP alleles in the stable chromatin binding mode (Fig 2C). Error bars denote s.e.m, n>26 cells per condition.

C FACS profile of propidium iodide stained SMC3-LAP cells in Fig 2F,G. Cells were synchronized in G2-phase and transfected with siRNA as indicated.

D Western blot showing HeLa cell extracts from Fig 2F,G after chromatin fractionation.

E Graph depicting normalized iFRAP intensity of SMC3-LAP(wt) after siRNA transfection and synchronization in G2-phase (Fig 2F,G). Error bars denote s.e.m, n>11 cells per condition.

F Graph depicting normalized iFRAP intensity of SMC3-LAP(QQ) after siRNA transfection and synchronization in G2-phase (Fig 2E,F). Error bars denote s.e.m, n>11 cells per condition.

G FACS profile of propidium iodide stained SMC3-LAP(wt) and (RR) cells after siRNA transfection and synchronization in G2-phase.

H Graph depicting normalized iFRAP intensity of SMC3-LAP(wt) and (RR) after siRNA transfection and synchronization in G2-phase. Error bars denote s.e.m, n=6 cells per condition.

I Quantification of stably chromatin bound SMC3-LAP after siRNA treatment. Error bars denote s.e.m, n=6 cells per condition.

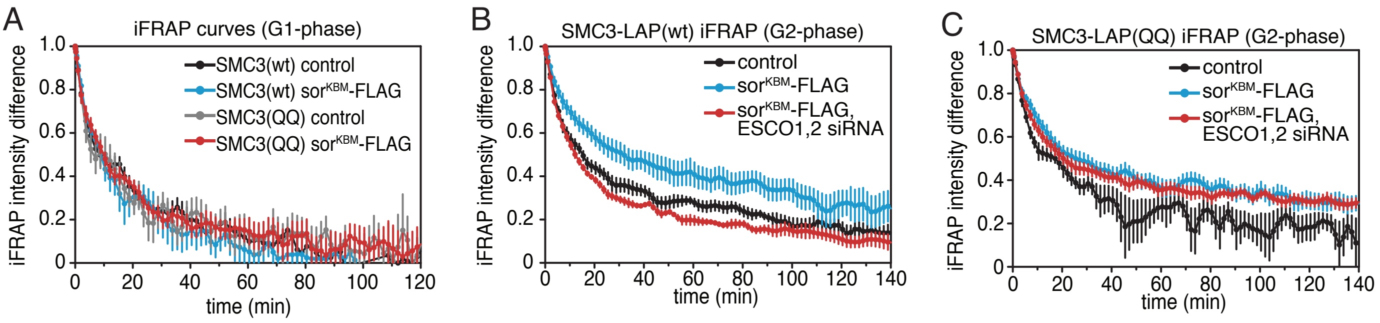

Appendix Figure S3. Sororin cannot stabilize cohesin–chromatin interactions before DNA replication (related to Fig 3)

A Graph depicting normalized iFRAP intensity of cells transfected with sororin^{KBM}-FLAG and synchronized in G1-phase (Fig 3D). Error bars denote s.e.m, n>15 cells per condition.

B Graph depicting normalized iFRAP intensity of SMC3-LAP(wt) cells transfected with sor^{KBM}-FLAG and siRNA as indicated and synchronized in G2-phase (Fig 3G). Error bars denote s.e.m, n>5 cells per condition.

C Graph depicting normalized iFRAP intensity of SMC3-LAP(QQ) cells transfected with sor^{KBM}-FLAG and siRNA as indicated and synchronized in G2-phase (Fig 3G). Error bars denote s.e.m, n>5 cells per condition.

Ladurner, Kreidl et al. Figure S4

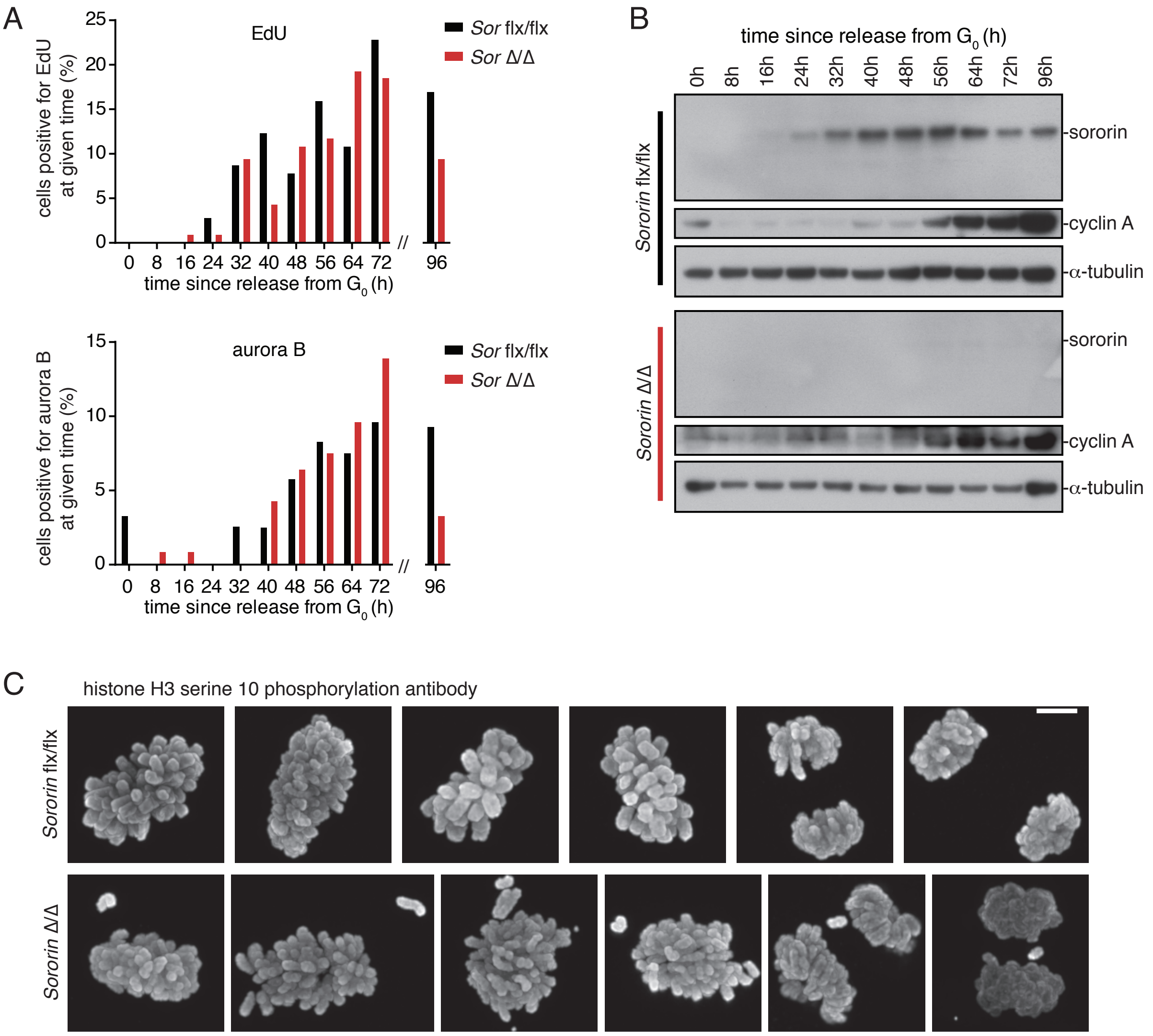

Appendix Figure S4. Sororin is dispensable for DNA replication and progression through interphase (related to Fig 5)

A Quantification of EdU-pulse labeling as marker for active DNA replication and aurora kinase B staining to mark G2/M-phase fibroblasts after *Cdca5* deletion and release into rich medium.

B Western blot showing lysates from cells released into rich medium after *Cdca5* deletion.

C Examples of mitotic cells with misaligned chromosomes after *Cdca5* deletion quantified in Figure 5H. Size bar, 5 μm.

Ladurner, Kreidl et al. Figure S5

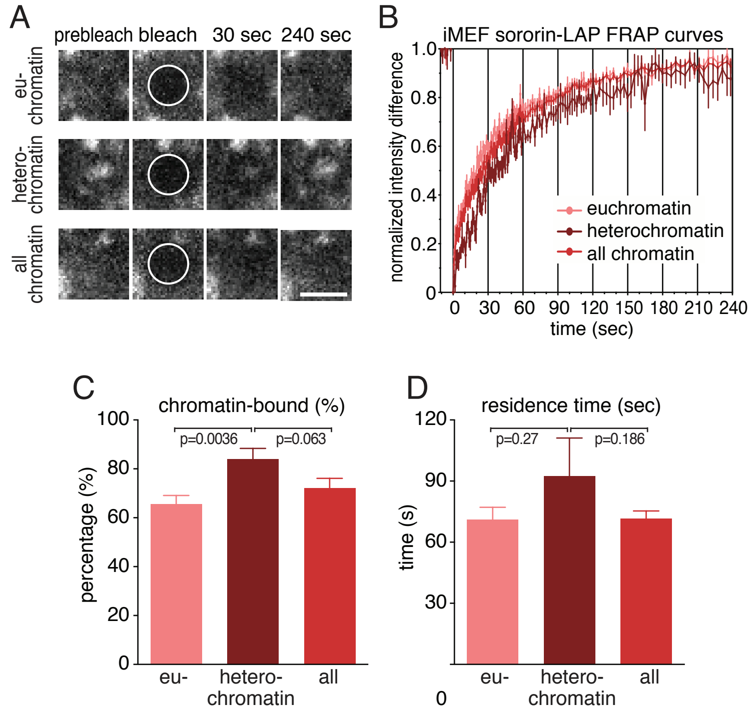

Appendix Figure S5. Sororin turns over rapidly on chromatin (related to Fig 7)

A–D Chromatin-bound sororin is enriched at heterochromatic regions in mouse fibroblasts.

A FRAP still images of sororin-LAP-AID nuclear regions. Data were divided into heterochromatin or euchromatin depending if the bleach area contained GFP signals at chromocenters or not (We named these regions ‘euchromatin’, although they presumably represent a mixture of euchromatin, facultative and non-pericentric heterochromatin). All chromatin corresponds to the dataset in Fig 7C-F. Size bar, 5 µm. Bleach area radius, 2 *µ*m.

B Graph depicting the normalized FRAP intensity to quantify turnover of sororin. Error bars denote s.e.m, n > 9 cells per condition.

C Quantification of the relative abundance of sororin-LAP-AID on chromatin. Error bars denote s.e.m., n > 9 cells per condition.

D Quantification of the residence time of sororin-LAP-AID on chromatin. Error bars denote s.e.m., n > 9 cells per condition.
