# Supplementary material for: Sororin actively maintains sister chromatid cohesion
Source: EMBO J. 2016 Feb 22;35(6):635–53. doi: 10.15252/embj.201592532 (PMC4801952; doi:10.15252/embj.201592532)
Supplement: Supplementary file 2 — Expanded View Figures PDF [file EMBJ-35-635-s002.pdf]

Expanded View Figures

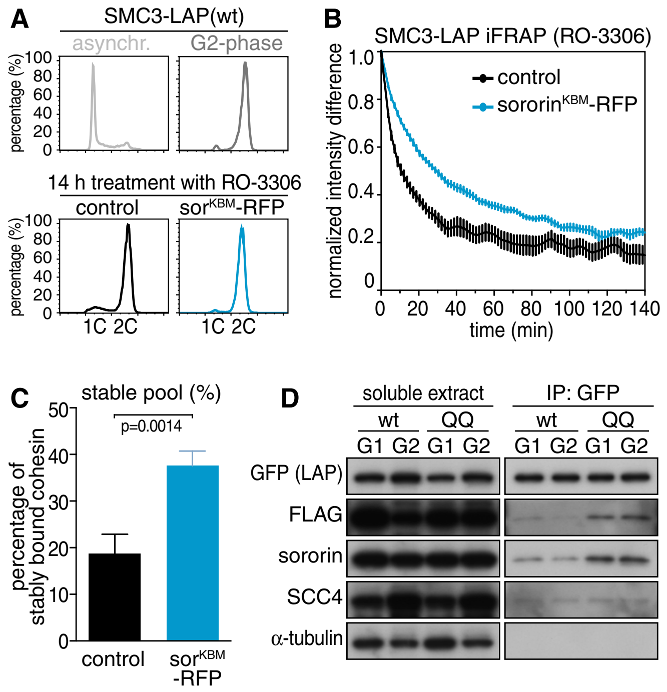

Figure EV1. Sororin cannot stabilize cohesin–chromatin interactions before DNA replication (related to Fig 3).

A FACS profile of propidium iodide-stained SMC3-LAP(wt) cells after double thymidine arrest, release into fresh medium for 6 h (G2-phase) and subsequent addition of RO-3306 (9 μM final conc.) to inhibit CDK1 activity. Sororin^{KBM}-RFP plasmid transfection was performed at the time of RO-3306 addition. Please note that the sororin^{KBM}-FLAG construct was modified so that the FLAG tag was replaced by monomeric red fluorescent protein (RFP).

B Graph depicting normalized iFRAP intensity of SMC3-LAP(wt) after prolonged G2-phase by CDK1 inhibition and transfection with plasmid as indicated. Error bars denote s.e.m., $n > 10$ cells per condition.

C Quantification of stably chromatin-bound SMC3-LAP in RO-3306-treated cells after transfection with sororin^{KBM}-RFP. Error bars denote s.e.m., $n > 10$ cells per condition. Unpaired *t*-test was used to compare conditions.

D Western blot showing fractionated soluble extracts after immunoprecipitation with GFP antibody. SMC3-LAP(wt) and (QQ) cells were transfected with sor^{KBM}-FLAG and synchronized in G1 or G2-phase.

 © 2016 The Authors

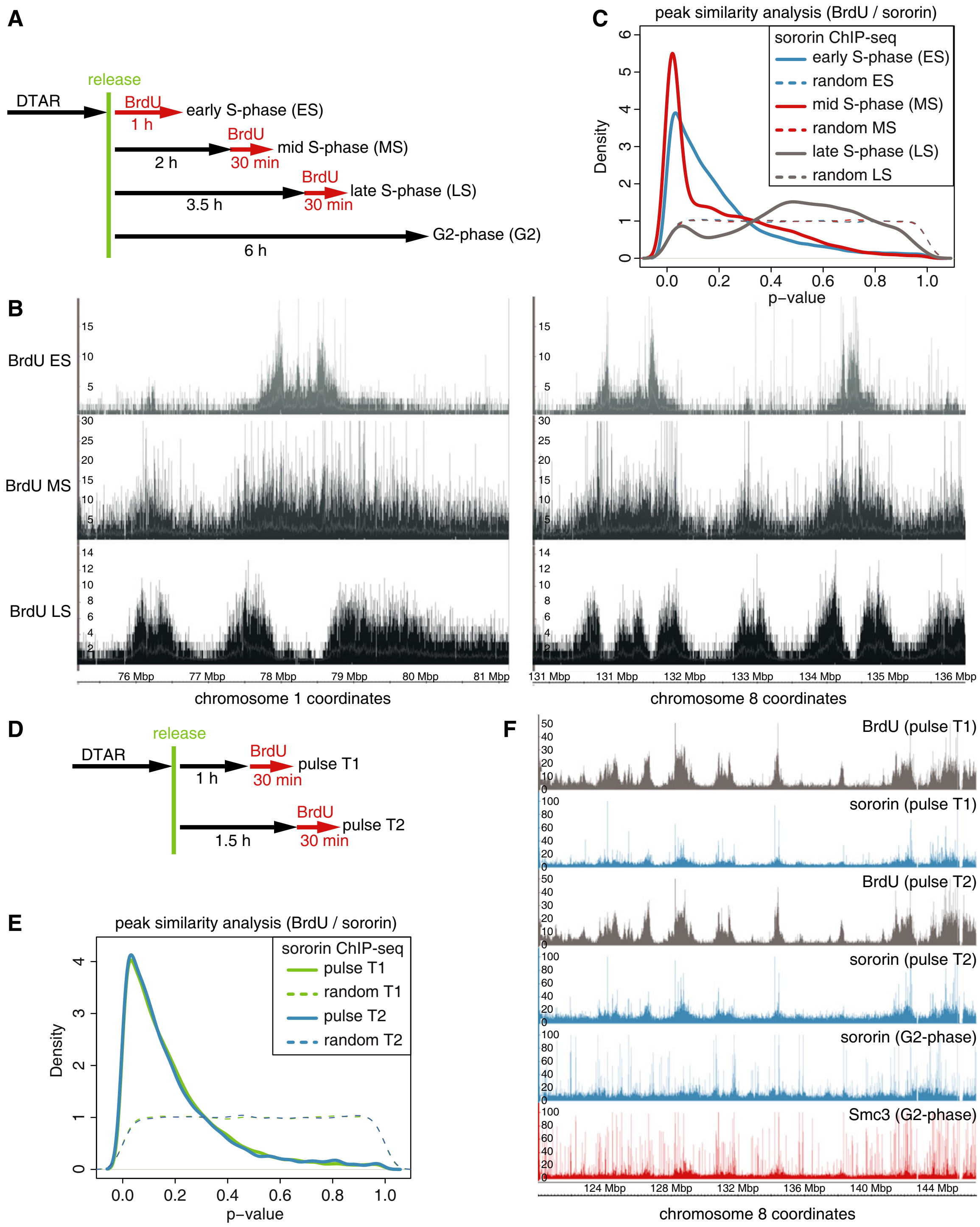

Figure EV2. The genome-wide association of sororin with cohesin occurs exclusively on replicated DNA (related to Fig 4).

A Experimental setup used to synchronize HeLa cells in different states of DNA replication after double thymidine arrest–release (DTAR).

B Examples of BrdU sequencing enrichment in early, mid-, and late S-phase. Please note the differences in *y*-axis scale between different time points. The varying sequencing enrichment possibly represents differences in population-wide replication dynamics.

C Quantification of the co-occupancy of BrdU incorporation and sororin localization in early, mid-, and late S-phase depicted as *P*-value distributions and compared to randomized controls. Please note that in late S-phase, the co-occupancy between sororin and BrdU is low, presumably because a large part of the genome was replicated and bound by sororin before the BrdU pulse.

D Experimental setup to synchronize HeLa cells at two time points corresponding to early S-phase.

E Graph depicting the co-occupancy of BrdU and sororin at S-phase time points t1 and t2.

F Examples of sororin and SMC3 ChIP-seq and BrdU DIP-seq data from early S-phase as compared to G2-phase.

© 2016 The Authors

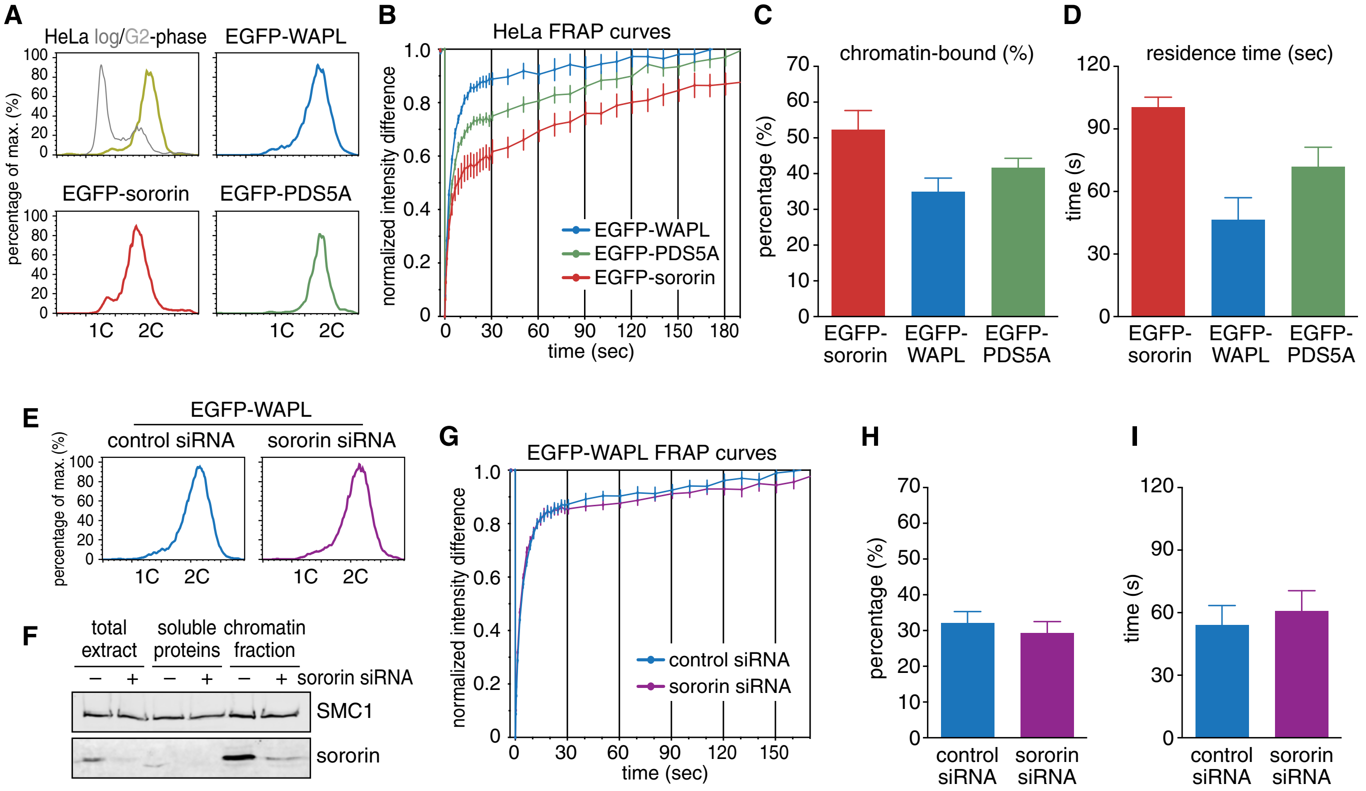

Figure EV3. Sororin, PDS5A, and WAPL turn over rapidly on chromatin (related to Fig 7).

A–D The regulatory cohesin binding proteins sororin, WAPL, and PDS5A associate dynamically with chromatin in HeLa cells. (A) FACS profile of propidium iodide-stained EGFP-sororin, EGFP-WAPL, and EGFP-PDS5A HeLa cells synchronized in G2-phase. Genes were homozygously tagged at endogenous loci by CRISPR-mediated homologous recombination. (B) FRAP profiles of sororin, WAPL, and PDS5A in G2-phase synchronized HeLa cells. Error bars denote s.e.m., n = 15 cells per condition. (C) Quantification of the relative abundance of sororin, WAPL, and PDS5A on chromatin as derived by curve fitting data in (B). Error bars denote s.e.m., n = 15 cells per condition. (D) Quantification of the mean residence time of sororin, WAPL, and PDS5A on chromatin. Error bars denote s.e.m., n = 15 cells per condition.

E–I Turnover of WAPL on chromatin in G2-phase is not detectably reduced by depletion of sororin. (E) FACS profiles of propidium iodide-stained EGFP-WAPL cells after siRNA transfection and synchronization in G2-phase. (F) Western blot showing whole-cell, soluble protein, and chromatin extracts of EGFP-WAPL HeLa cells after treatment with sororin or control siRNAs. SMC1 levels were used as a loading control. (G) FRAP profiles of EGFP-WAPL HeLa cells in G2-phase after siRNA treatment. Error bars denote s.e.m., $n > 14$. (H) Quantification of the relative abundance of WAPL on chromatin after sororin RNAi. Error bars denote s.e.m., $n > 14$. (I) Quantification of the chromatin residence time of WAPL after sororin RNAi. Error bars denote s.e.m., $n > 14$.

 © 2016 The Authors

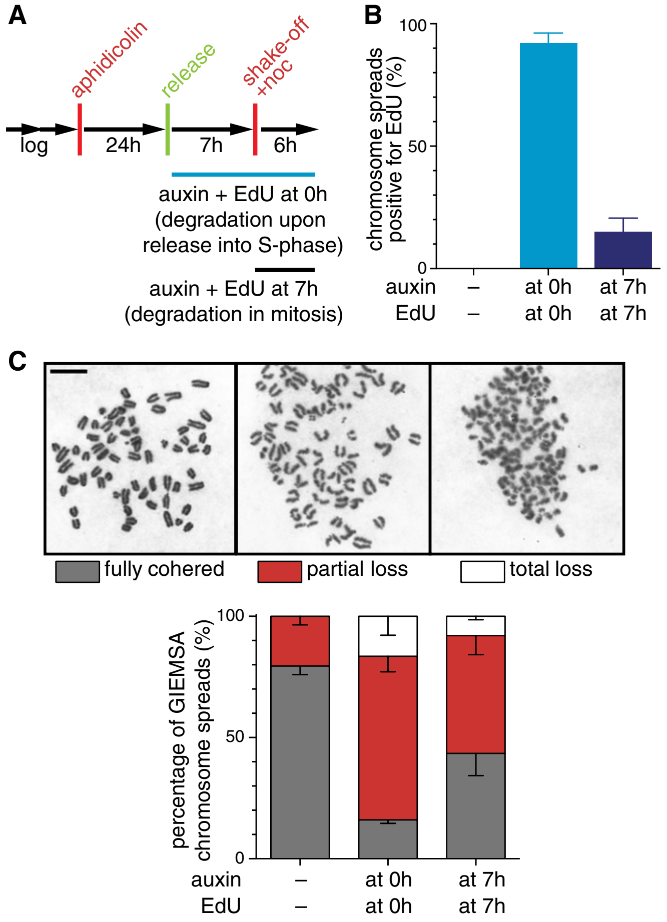

Figure EV4. Sororin is a cohesion maintenance factor (related to Fig 8).

A Experimental setup used for cell synchronization, auxin, and EdU treatment of *Cdca5* Δ/Δ sor-LAP-AID F-box mouse embryonic fibroblasts. Shake-off was used to enrich for mitotic cells.

B Quantification of EdU-labeled prometaphase cells after treating cells with auxin and EdU at the indicated time points. Please note that although shake-off was used to enrich for mitotic cells, 15% of cells still incorporated EdU in this population. Error bars denote s.d., n = 200 cells per condition.

C Examples and analysis of chromosome spreads after auxin treatment and Giemsa staining. Scale bar, 10 μm. Error bars denote s.d., n = 200 cells per condition.
